# Urinary metabolomic signatures in reticular oral lichen planus

**DOI:** 10.1016/j.heliyon.2020.e04041

**Published:** 2020-05-22

**Authors:** Xu-yan Yang, Xu-zhao Li, Shuai-nan Zhang

**Affiliations:** a First Affiliated Hospital, Heilongjiang University of Chinese Medicine, Harbin 150040, PR China; b College of Pharmacy, Guizhou University of Traditional Chinese Medicine, Guian new area 550025, PR China

**Keywords:** Biochemistry, Immunology, Pathology, Oral medicine, Health technology, Diagnostics, Biomarkers, Oral lichen planus, Reticular type, Urinary metabolomic signatures

## Abstract

Oral lichen planus (OLP) is a chronic inflammatory disease. Among all the clinical forms in OLP, reticular type has the highest incidence rate. Previous studies have applied metabolomics to investigate the metabolic changes of oral mucosa and blood samples from reticular OLP patients. Urinary metabolomic signatures is also useful in analyzing the pathological changes of the patients, which was a complement to the previous studies. Through these researches, we may have a more comprehensive understanding of the disease. Metabolic profiles of urinary samples from OLP patients and control subjects were analyzed by liquid chromatography (LC)-mass spectrometry (MS) system. Differentially expressed metabolites were identified via OSI/SMMS software for the pathology analysis. Totally, 30 differentially expressed metabolites were identified. Pathological network showed that these metabolites participated in 8 pathological processes, that is, DNA damage and repair disorder, apoptosis process, inflammatory lesion, oxidative stress injury, carbohydrate metabolism disorder, mood dysfunction, abnormal energy expenditure, and other pathological process. These findings demonstrated that the analysis of human urine metabolome might be conducive to the achievement of the objectives of this study.

## 1 Introduction

Oral lichen planus (OLP) is a chronic inflammatory disease [1, 2, 3]. In addition to the local damage to the oral mucosa [4], the disease may be also associated with the pathological changes in other parts of the body [1, 2]. The morbidity rate of OLP is between 0.5% and 3%, 0.5–12.5% of which can encounter malignant transformation [1]. Women are the main affected population of the disease, whose incidence rate is more than twice as men in the age range between 30 and 60 years [5]. In clinical practice, OLP is classified into 6 clinical forms (reticular, erosive, papular, plaque-like, atrophic, and bullous). Among them, reticular type has the highest incidence rate, researches on which may benefit more patients.

Previous studies have used metabolomics to analyze the biochemical changes in the oral lesion tissues and the blood system of reticular OLP patients [1, 4]. In addition to oral tissues and blood samples, urine samples can also be used for oral disease research [3]. Through the study of these three biological samples, it is possible to have a more comprehensive understanding of the pathogenesis of the disease. Thus, this experiment would be a complement to the previous studies and applied metabolomics to investigate the metabolic changes of urine samples from reticular OLP patients.

## 2 Methods

### 2.1 Chemicals and reagents

Acetonitrile, formic acid, methanol, and deionized water were purchased from CNW Technologies GmbH (Düsseldorf, Germany). L-2-Chlorophenylalanine was from Shanghai Hengchuang Bio-technology Co., Ltd. (Shanghai, PR China). All chemicals and reagents were analytical or HPLC grade.

### 2.2 Samples collection and preparation

**Figure 1:**
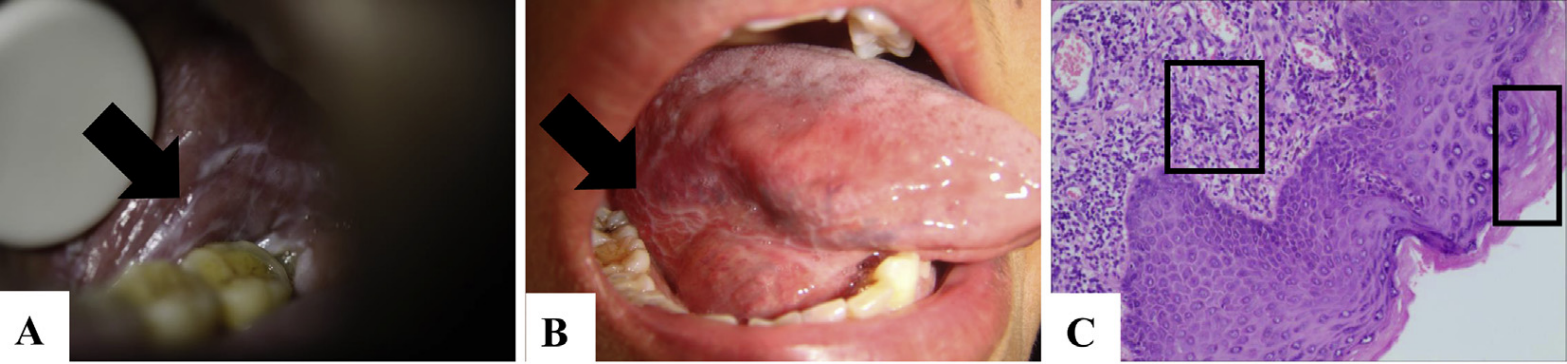
(A and B) Clinical pictures and (C) histopathological image of reticular OLP patient. (A and B) White reticular striae were found on the surface of oral mucosa and tongue (indicated by an arrow). (C) Parakeratosis and numerous inflammatory cells were found in histopathological image (magnification×400). These are the characteristic features of reticular OLP. The images were published in our previous study [4].

All subjects provided the signed informed-consent documents. The research protocol was reviewed by the Ethical Committees of First Affiliated Hospital of Heilongjiang University of Chinese Medicine (date of permission: December 29, 2014, permission No. HZYLLKY201400601) and was executed in agreement with the principles outlined in the Declaration of Helsinki. Urinary samples were collected from 16 reticular OLP patients and 24 control subjects between January 1, 2016 and June 30, 2016 at the First Affiliated Hospital of Heilongjiang University of Chinese Medicine, PR China [1, 4]. The reticular OLP patients consisted of 10 female and 6 male and their age ranged from 20 to 54 years. The control subjects consisted of 15 female and 9 male and their age ranged from 18 to 48 years. Reticular OLP patients were diagnosed in according with the clinical and pathological criteria (Figure 1) [6]. The subjects did not have other diseases, such as diabetes, hypertension, kidney disease, cardiovascular diseases, and other intraoral inflammation, etc. All subjects were kept on a normal diet and did not experience any treatment prior to admission, and the samples were taken at their initial visit.

150 μl of urine sample was centrifuged at 13,000 rpm, 4 °C for 10 min. 100 μl of supernatant and 10 μl of internal standard (L-2-chlorophenylalanine in methanol, 0.3 mg/ml) were added to a centrifuge tube and vortexed for 10 s. Afterwards, 100 μl of ice-cold mixture methanol-acetonitrile (2:1, v/v) was added, and the mixture was vortexed for 1 min, ultrasound-extracted in an ice-bath for 10 min, stored at -20 °C for 30 min. The extraction was centrifuged at 13,000 rpm, 4 °C for 15 min. 160 μl of supernatant from each tube was filtered through a sterile microfilter (0.22 μm) and transferred to the vial. The vial was stored at -80 °C until liquid chromatography (LC)-mass spectrometry (MS) analysis. Quality control (QC) samples were prepared by mixing equal volume of all samples.

### 2.3 LC-MS analysis

LC analysis was undertaken on an Ultimate 3000 RS UHPLC system (Thermo Fisher Scientific). A 5μl aliquot of prepared sample was inserted into an Acquity UPLC BEH C_18_ column (1.7 μm, 2.1 × 100 mm, Waters Corp). The column temperature was 45 °C, and the sample temperature was kept at 4 °C. The mobile phases consisted of (A) water (containing 0.1 % formic acid, v/v) and (B) acetonitrile (containing 0.1 % formic acid, v/v). The LC separation was achieved under the following gradient conditions: 0–1 min holding at 1 % B, 1–20 % B over 1–6.5 min, 20–30 % B over 6.5–8.5 min, 30–35 % B over 8.5–13 min, 35–70 % B over 13–14 min, 70–99 % B over 14–16.5 min, 16.5–17.5 min holding at 99 % B, then 17.5–18.5 min, 99 %–1 % B, and 18.5–19.5 min holding at 1 % B. The flow rate was 0.4 ml/min.

MS analysis was conducted on a quadrupole-Orbitrap mass spectrometer (Q-Exactive, Thermo Fisher Scientific) equipped with a heated electrospray ionization source working in both positive and negative ion modes. The mass range was from m/z 66.7 to 1,000.5. The resolution was set at 70,000 for the full MS scans and 35,000 for HCD MS/MS scans. The Collision energy was set at 10, 20, and 40 eV. The mass spectrometer operated as follows: spray voltage, 3,000 V (positive) and 2,500 V (negative); sheath gas flow rate, 45 arbitrary units; auxiliary gas flow rate, 15 arbitrary units; capillary temperature, 350 °C.

To guarantee the stability and reproducibility of the LC-MS analysis, a QC sample, pooled with equal aliquots of each individual sample, was inserted after every 10 samples throughout the study.

### 2.4 Multivariate data analysis

The data processing step was achieved using the XCMS software (1.50.1 version), which generated a matrix of features consisted of chromatographic, retention time, exact mass, and ion intensity. Subsequently, the processed data from both modes was united to be a combine data set and imputed into the SIMCA software package (version 14.0). Principle component analysis (PCA) and orthogonal partial least-squares-discriminant analysis (OPLS-DA) were performed by SIMCA software package. PCA was carried out to preliminarily find out their metabolic distinction. Then OPLS-DA was applied to sharpen an established separation between both groups of observations plotted in PCA, and improve differentially expressed metabolites discovery efforts. Variable importance in the projection (VIP) ranks the overall contribution of each variable to the OPLS-DA model. Student's t-test was applied for the statistical analysis and carried out with the R Language (version 3.3.1). Differentially expressed metabolites were selected according to their variable VIP statistics (VIP>2) and predefined P-value thresholds (*p* < 0.05).

### 2.5 Differentially expressed metabolites identification and pathological network construction

Based on the precursor ion and MS/MS information matching, differentially expressed metabolites identification was carried out by OSI/SMMS software (Dalian Institute of Chemical Physics, Chinese Academy of Sciences and Dalian ChemData Solution Information Technology Co., Ltd, PR China). Reference material database (Dalian Institute of Chemical Physics, Chinese Academy of Sciences and Dalian ChemData Solution Information Technology Co., Ltd, PR China), HMDB and, METLIN were used as the database source. Pathological network was constructed by Cytoscape software (version 3.6.0).

## 3 Results

### 3.1 Metabolomics analysis and differentially expressed metabolites identification

Through metabolomics analysis, 6391 metabolite ions (2830 in positive ion mode, 3561 in negative ion mode) were detected in urinary samples. All ions were normalized to the total peak area of each sample to get a minimum relative standard deviation (RSD). 93.22 % of ions (2638) in positive ion mode and 98.29 % (3500) in negative ion mode displayed less than 30 % of RSD, which showed the good reproducibility of the metabolomics method and were used for the further data processing.

**Figure 2:**
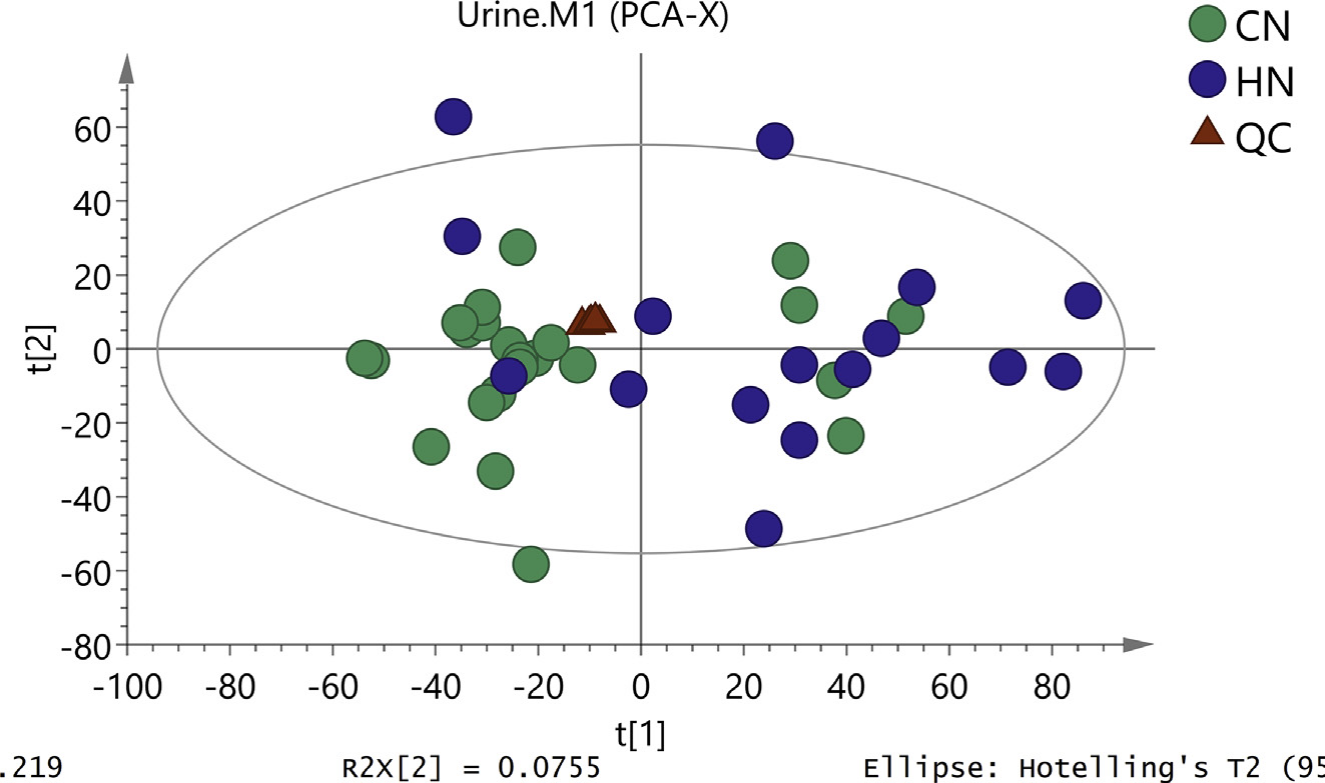
PCA score plot based on the oral mucosal metabolic profiling of (CZ) control and (HZ) reticular OLP groups.

**Figure 3:**
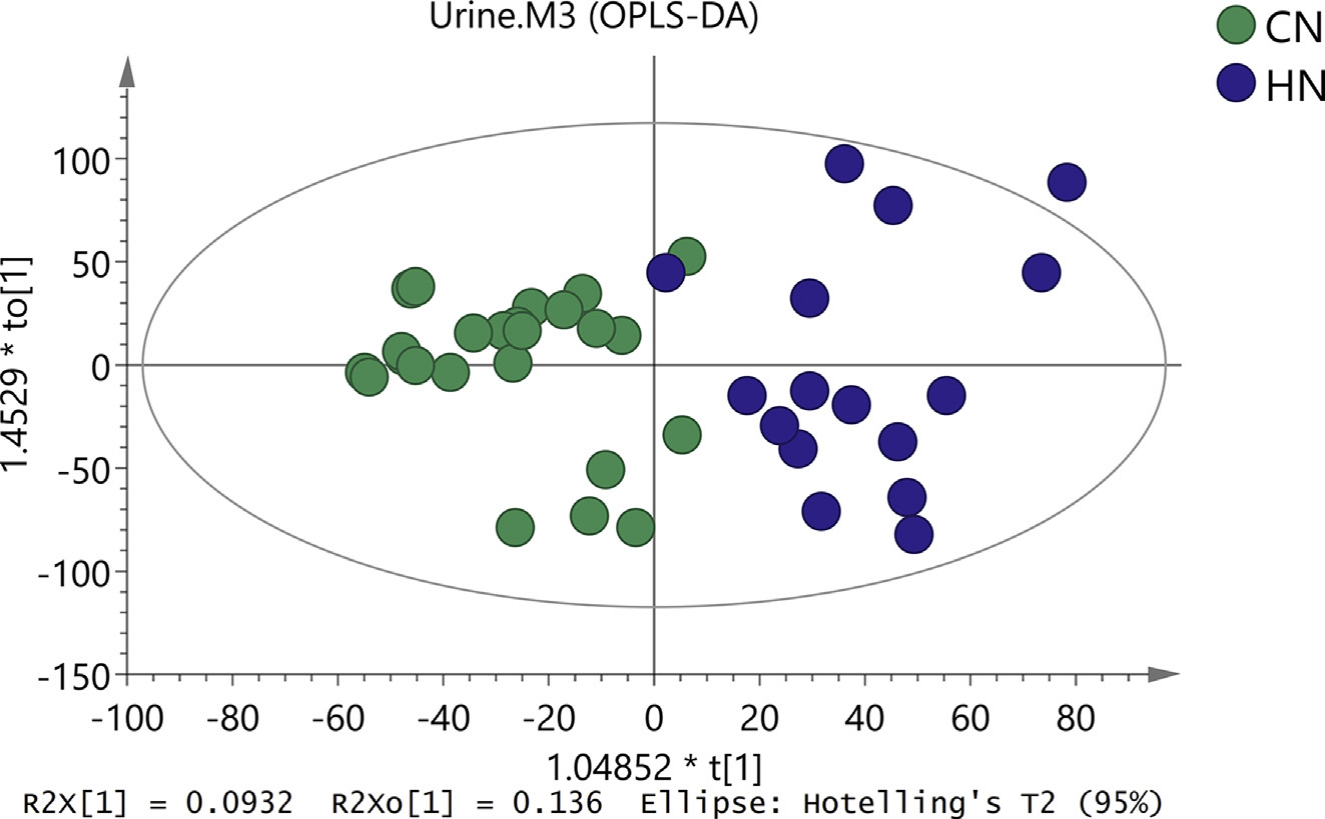
OPLS-DA score plot based on the oral mucosal metabolic profiling of (CZ) control and (HZ) reticular OLP groups.

Clustering of the QC samples was investigated by PCA to reveal the platform stability. QC samples were tightly clustered, demonstrating good reproducibility of the data (Figure 2). PCA showed that the metabolic profile was different between both groups (Figure 2), while in OPLS-DA, the metabolic difference between them was more obvious (Figure 3). The result of OLPS-DA showed the significant biochemical perturbation in urinary samples from the patients.

30 differentially expressed metabolites were identified by the analysis of OSI/SMMS software (Table 1). Compared to the control group, the levels of 13 metabolites were up-regulated, and 17 metabolites were down-regulated in reticular OLP group.

**Table 1:** Differentially expressed metabolites identified of reticular OLP patients.

NO.	Metabolites	VIP	RT (min)	m/z	Adduct	Delta (ppm)	Fold change (OLP/Control)	Ion mode	Database
1	Pro-Leu	8.70	1.01	229.15435	M + H	0	0.76	Pos	RMD
2	Oxalacetic acid	7.93	19.82	154.99020	M + Na	32	1.39	Pos	HMDB
3	Histidinol	6.63	17.71	124.08697	M + H–H_2_O	4	1.50	Pos	METLIN
4	Aminoacetone	6.50	19.18	112.01847	M + K	23	1.41	Pos	HMDB
5	N,N-Dimethylaniline	6.07	18.88	122.09663	M + H	2	1.37	Pos	METLIN
6	13-cis-Retinal	5.59	11.40	302.23280	M + NH_4_	53	0.46	Pos	METLIN
7	Kynurenic acid	4.82	5.17	190.04971	M + H	1	0.53	Pos	HMDB
8	5-Aminopentanoic acid	4.69	3.32	100.07608	M + H–H_2_O	1	0.34	Pos	METLIN
9	Isobutyryl carnitine	4.56	4.38	232.15404	M + H	1	0.62	Pos	METLIN
10	Methylarsonite	4.29	19.17	141.98332	M + NH_4_	7	1.41	Pos	HMDB
11	Ornithine	3.82	18.87	150.12767	M + NH_4_	30	1.37	Pos	METLIN
12	3,4-Dihydroxymandelic acid	3.40	19.18	207.01750	M + Na	48	1.42	Pos	HMDB
13	Chlorate	3.23	19.39	117.92680	M + Cl	32	1.66	Neg	HMDB
14	Hexadecanamide	2.89	17.48	256.26308	M + H	5	1.40	Pos	RMD
15	Ala-His	2.83	13.55	209.11699	M + H–H_2_O	58	0.61	Pos	METLIN
16	Arg-Val	2.70	4.37	312.13042	M + K	47	0.73	Pos	METLIN
17	Succinic acid	2.68	19.20	156.99066	M + K	6	1.40	Pos	HMDB
18	Anthranilic acid	2.57	4.17	120.04427	M + H–H_2_O	5	0.65	Pos	METLIN
19	Asn-Thr	2.57	13.56	251.12757	M + NH_4_	32	0.60	Pos	METLIN
20	p-CHLOROPHENYLALANINE	2.50	4.93	198.03115	M-H	8	2.32	Neg	METLIN
21	Methoxyacetic acid	2.43	19.18	113.01695	M + Na	35	1.41	Pos	HMDB
22	D-(-)-Lyxose	2.43	3.67	151.06144	M + H	9	0.72	Pos	METLIN
23	Carnosine	2.41	0.75	227.12429	M + H	46	0.71	Pos	METLIN
24	Trp-Val	2.40	5.10	304.16481	M + H	2	0.58	Pos	HMDB
25	Arg-Ile	2.18	9.80	288.21607	M + H	45	0.44	Pos	METLIN
26	2-Oxo-4-methylthiobutanoic acid	2.12	19.16	186.98107	M + K	8	1.38	Pos	HMDB
27	Phe-Asp	2.07	5.31	281.10975	M + H	12	0.56	Pos	HMDB
28	2-trans,4-cis-Decadienoylcarnitine	2.03	9.99	312.21395	M + H	10	0.48	Pos	HMDB
29	9-Decenoylcarnitine	2.02	11.03	336.21626	M + Na	5	0.49	Pos	HMDB
30	Arg-Thr	2.01	6.56	258.16996	M + H–H_2_O	48	0.47	Pos	METLIN

Delta: ppm error between the observed mass and the theoretical mass.

Fold change: the ratio of the average level of metabolites in reticular OLP group to that in control group.

The metabolites were detected in (POS) positive and (NEG) negative ESI mode.

RMD: reference material database.

### 3.2 Pathological network construction

**Figure 4:**
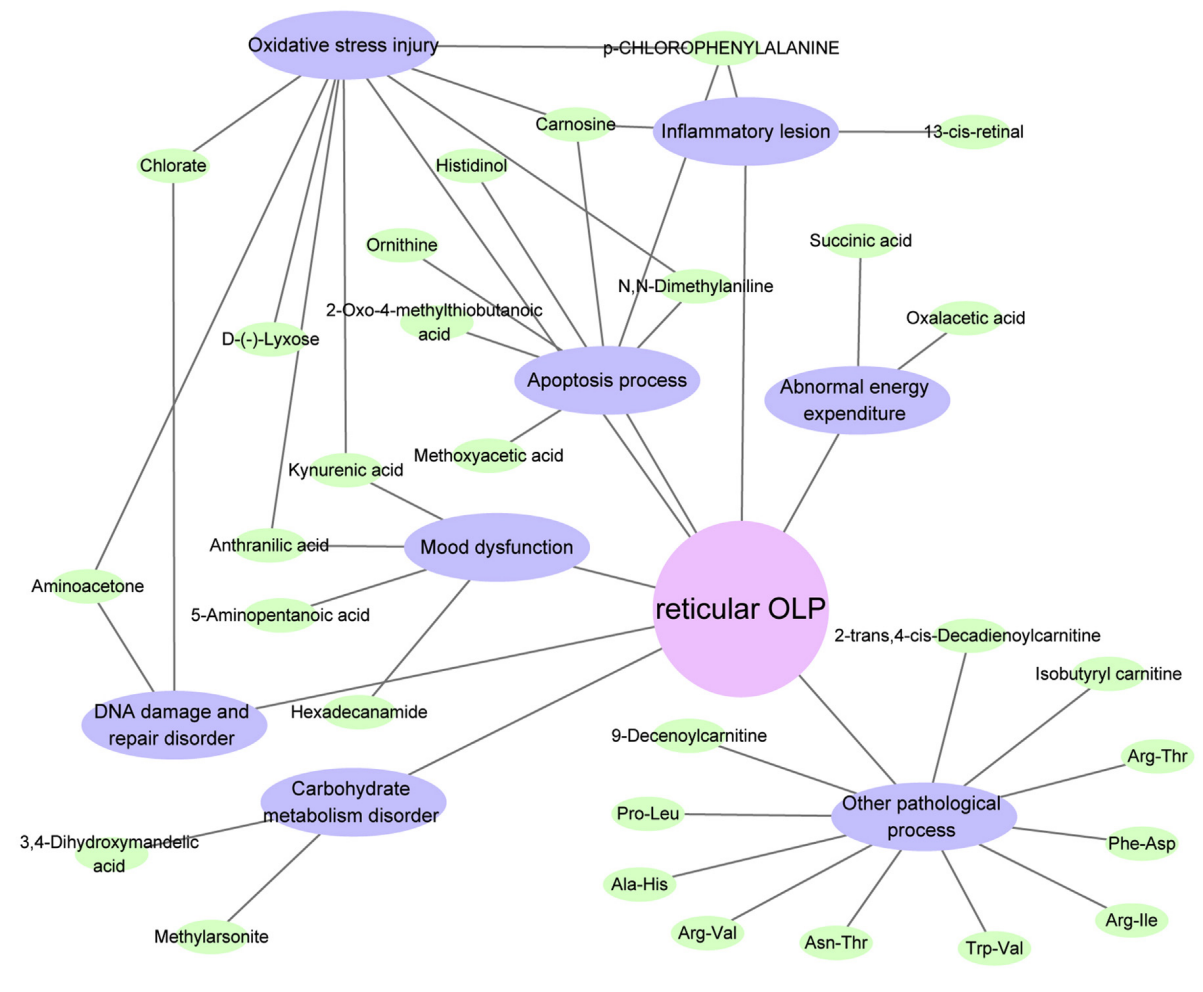
Pathological network associated with reticular OLP. Blue nodes represent pathological processes, and green nodes represent metabolites.

Pathological network displayed in Figure 4 shows that these metabolites were involved in 8 pathological processes, that is, DNA damage and repair disorder, apoptosis process, inflammatory lesion, oxidative stress injury, carbohydrate metabolism disorder, mood dysfunction, abnormal energy expenditure, and other pathological process.

## 4 Discussion

In the previous researches, we have investigated the metabolic profiling in the oral lesion mucosa and serum of reticular OLP [1, 4]. This experiment discussed the urinary metabolomic signatures in reticular OLP. Metabolism and excretion processes of the endogenous metabolites are ongoing all the time. Endogenous metabolites that are excreted into the urine originally existed in the internal environment, which had the effects on the body as well. Therefore, the studies on the metabolites in urine can be used to reversely analyze the possible pathological changes in the patients to some extent, which are important for understanding the pathogenesis of the diseases. The metabolic information obtained from urinary sample may affect 7 pathological processes that are also described in the previous studies [1, 4], that is, DNA damage and repair disorder, apoptosis process, inflammatory lesion, oxidative stress injury, Carbohydrate metabolism disorder, Mood dysfunction, and abnormal energy expenditure. Meanwhile, urinary metabolic information also reflects other pathological processes in reticular OLP.

### 4.1 DNA damage and repair disorder in reticular OLP

DNA damage occurs frequently in OLP patients, which is one of the important pathological features of the disease [7]. The expression of aminoacetone was enhanced in the urine of patients with OLP, an increase in whose concentration results in DNA cleavage [8]. Additionally, the level of chlorate was increased in the urine sample as well, which induces DNA damage and DNA-protein cross-linking [9]. Therefore, we can observe that the increase in the concentrations of both metabolites might be one of the important inducing factors of DNA damage in OLP.

### 4.2 Apoptosis process in reticular OLP

Apoptotic cell death is a key cause of oral and blood cells destruction in OLP [10, 11]. In the urine samples from OLP patients, 6 metabolite levels were up-regulated and one was down-regulated, whose dysregulations are closely related to the apoptosis process. Histidinol can be converted into histidine [12], whose up-regulation is beneficial to the formation of histidine, as shown in the results of our previous research [4]. Accompanied with oxidative stress injury, histidine can cause DNA double-strand breaks, and eventually lead to cell apoptosis [13]. In addition, according to the calculation of SIMCOMP2 (http://www.genome. jp/tools/simcomp2/), the molecular structures of both metabolites had a relatively high similarity (similarity score = 0.84). Therefore, according to the theory “similarity often leads to analogies” [14], we concluded that histidinol itself might also produce the biological activities similar to histidine. N,N-Dimethylaniline is a substituted derivative of aniline. Through the calculation of SIMCOMP2, the molecular structures of both metabolites were also similar (similarity score = 0.76). Thus, we inferred that N,N-dimethylaniline might also induce apoptosis similar to aniline [15]. The up-regulation of ornithine may induce apoptosis via regulating the level of cell cycle proteins [16]. Our previous study has shown that the level of p-chlorophenylalanine in the oral mucosa is reduced [4], but in the present experiment, that in the urine was increased. The cause of the difference might be that OLP accelerated its excretion into the urine, subsequently resulting in a decrease in its content in internal milieu. The reduction of p-chlorophenylalanine level may induce apoptosis through enhance the expression of serotonin [17, 18]. Methoxyacetic acid is a major metabolite of ethylene glycol monomethyl ether, which may induce cell apoptosis via its interactions with protein kinase [19]. 2-Oxo-4-methylthiobutanoic acid is the direct precursor of methional, which is a potent inducer of apoptosis [20]. Its up-regulation might lead to the increased production of methional, and eventually induce the cell apoptosis. Carnosine is an anti-apoptotic metabolite, whose deficiency might weaken the protective effects against the apoptosis [21].

### 4.3 Inflammatory lesion in reticular OLP

The pro-inflammatory mechanisms are activated in both oral tissues and circulatory system of OLP patients [22, 23]. 13-cis-Retinal and carnosine can exert their anti-inflammatory effects in organisms [24, 25], whose deficiencies might not be conducive to the recovery of inflammatory lesion in the disease. In addition, the down-regulation of internal p-chlorophenylalanine by its increased urinary excretion can enhance serotonin expression [4, 18], which may also cause inflammatory injury subsequently [26].

### 4.4 Oxidative stress injury in reticular OLP

Oxidative stress injury exists in both oral mucosa and serum of OLP patients [4, 27]. In addition to the induction of DNA damage, the increased concentrations of aminoacetone and chlorate may also lead to oxidative stress injury [28, 29]. From the above description we can observe that N,N-dimethylaniline might produce the biological effects similar to aniline, whose up-regulation might trigger oxidative stress as well [15]. Anthranilic acid, kynurenic acid, carnosine, and D-(-)-lyxose have antioxidant activities [21, 24, 30, 31, 32], whose deficiencies might not be conducive to the recovery of oxidative stress damage in the disease. Additionally, a deficiency of p-chlorophenylalanine in the internal milieu might also participate in the process of oxidative stress by increasing serotonin level [4, 33].

### 4.5 Carbohydrate metabolism disorder in reticular OLP

Abnormal carbohydrate metabolism may influence oral epithelial cell membrane of OLP patients [34, 35]. 3,4-Dihydroxymandelic acid is a major metabolite of the catecholamines, epinephrine and norepinephrine [36]. The up-regulation of 3,4-dihydroxymandelic acid might mean an increase in the release of these two catecholamines. As shown in the previous studies [37, 38], psychological stress state may induce the release of both catecholamines in OLP patients, whose up-regulations can inhibit insulin release [39, 40]. In addition, methylarsonite may also impair glucose-stimulated insulin secretion through the inhibition of mitochondrial respiration and calcium influx [41]. A deficiency of the hormone may trigger carbohydrate dysregulation [42]. From these point of view, we may know that insulin might act as a bridge to link both metabolites and carbohydrate metabolism disorder.

### 4.6 Mood dysfunction in reticular OLP

Mood dysfunction, such as depression, anxiety, and stress, plays the important roles in OLP patients [43, 44]. Anthranilic acid and kynurenic acid are the metabolites of tryptophan. Our previous study has shown that tryptophan level is down-regulated in the blood of OLP patients [1], which may lead to the decrease in the production of both metabolites, as shown in Table 1. The deficiency of tryptophan *in vivo* is closely related to mood dysfunction [45]. 5-Aminopentanoic acid is a lysine degradation product. The down-regulation of 5-aminopentanoic acid might mean the deficiency of lysine *in vivo*, which would increase stress-induced anxiety [46]. In the current experiment, the urinary excretion pattern of hexadecanamide was similar to that of p-chlorophenylalanine. The disease might also accelerate the excretion of hexadecanamide into the urine, and then leading to its down-regulation in the blood [1]. Our previous research has shown that hexadecanamide might have a sleep-inducing action similar to oleamide, whose deficiency might further affect the mood states in OLP patients [1, 47, 48].

### 4.7 Abnormal energy expenditure in reticular OLP

Our previous study has showed that abnormal energy expenditure also appears in OLP [4]. Oxalacetic acid is an intermediate of the citrate cycle, and reacts with Acetyl-CoA to form citrate [49]. Succinic acid, a component of the citrate cycle, can donate electrons to the electron transfer chain. Therefore, the overexpression of both metabolites might increase energy metabolism through the activation of the citrate cycle, so as to meet the increasing demand for energy in some pathological processes of OLP.

### 4.8 Other pathological process in reticular OLP

In the current research, reticular OLP also affected the levels of 3 carnitines and 8 dipeptides in the urine samples. However, the correlation between reticular OLP and the pathological processes induced by these metabolites was still unknown. In the further studies, the pathological effects of these metabolites on OLP also need more attention.

In summary, the research fully demonstrated that urinary metabolomics was useful for the study on the pathogenesis of reticular OLP, which also echoed the results of the previous oral mucosa and serum metabolomics analyses to some extent. Additionally, in the present research, we also obtained the additional information that was unavailable in the previous metabolomics analyses. This experiment was a complement to the previous studies. Through these researches, we may have a more comprehensive understanding of the disease.

However, there are also limitations to the present study. Metabolic changes in urine do not yet reflect the malignant potential of the disease. Therefore, in the follow-up study, the malignant mechanism of the disease is worthy of further study.

## Declarations

### Author contribution statement

X. Yang: Conceived and designed the experiments; Analyzed and interpreted the data.

X. Li: Conceived and designed the experiments; Performed the experiments; Analyzed and interpreted the data; Wrote the paper.

S. Zhang: Conceived and designed the experiments; Wrote the paper.

### Funding statement

X. Yang was spported by 10.13039/100014717National Outstanding Youth Science Fund Project of National Natural Science Foundation of China (81403441).

### Competing interest statement

The authors declare no conflict of interest.

### Additional information

No additional information is available for this paper.

## References

[1] Li X.Z., Zhang S.Z., Yang X.Y. (2019). Serum-based metabolomics characterization of patients with reticular oral lichen planus. Arch. Oral Biol..

[2] Yang X.Y., Zhang S.N., Li X.Z., Wang Y., Yin X.D. (2017). Analysis of human serum metabolome for potential biomarkers identification of erosive oral lichen planus. Clin. Chim. Acta.

[3] Li X.Z., Yang X.Y., Wang Y., Zhang S.N., Zou W., Wang Y., Li X.N., Wang L.S., Zhang Z.G., Xie L.Z. (2017). Urine metabolic profiling for the pathogenesis research of erosive oral lichen planus. Arch. Oral Biol..

[4] Yang X.Y., Li X.Z., Zhang S.Z. (2018). Metabolomics analysis of oral mucosa reveals profile perturbation in reticular oral lichen planus. Clin. Chim. Acta.

[5] Carbone M., Arduino P.G., Carrozzo M., Gandolfo S., Argiolas M.R., Bertolusso G., Conrotto D., Pentenero M., Broccoletti R. (2009). Course of oral lichen planus: a retrospective study of 808 northern Italian patients. Oral Dis..

[6] van der Meij E.H., Schepman K.P., van der Waal I. (2003). The possible premalignant character of oral lichen planus and oral lichenoid lesions: a prospective study. Oral Surg. Oral Med. Oral Pathol. Oral Radiol. Endod..

[7] Dillenburg C.S., Martins M.A., Almeida L.O., Meurer L., Squarize C.H., Martins M.D., Castilho R.M. (2015). Epigenetic modifications and accumulation of DNA double-strand breaks in oral lichen planus lesions presenting poor response to therapy. Medicine.

[8] Hiraku Y., Sugimoto J., Yamaguchi T., Kawanishi S. (1999). Oxidative DNA damage induced by aminoacetone, an amino acid metabolite. Arch. Biochem. Biophys..

[9] Ali S.N., Ansari F.A., Arif H., Mahmood R. (2017). Sodium chlorate induces DNA damage and DNA-protein cross-linking in rat intestine: a dose dependent study. Chemosphere.

[10] Sugerman P.B., Savage N.W., Walsh L.J., Zhao Z.Z., Zhou X.J., Khan A., Seymour G.J., Bigby M. (2002). The pathogenesis of oral lichen planus. Crit. Rev. Oral Biol. Med.: Off. Publ. Am. Assoc. Oral Biol..

[11] Neppelberg E., Johannessen A.C., Jonsson R. (2001). Apoptosis in oral lichen planus. Eur. J. Oral Sci..

[12] Ames B.N. (1957). The biosynthesis of histidine; L-histidinol phosphate phosphatase. J. Biol. Chem..

[13] Palomba L., Brambilla L., Brandi G., Sestili P., Cattabeni F., Cantoni O. (1996). Low levels of hydrogen peroxide and L-histidine induce DNA double-strand breakage and apoptosis. Eur. J. Pharmacol..

[14] Klopmand G. (1992). Concepts and applications of molecular similarity. J. Comput. Chem..

[15] Wang Y., Gao H., Na X.L., Dong S.Y., Dong H.W., Yu J., Jia L., Wu Y.H. (2016). Aniline induces oxidative stress and apoptosis of primary cultured hepatocytes. Int. J. Environ. Res. Publ. Health.

[16] Zhang J.H., Lu Q., Shi W.J., Wu Z.Z., Wang L.S. (2005). The induction apoptosis of HL-60 cells by low molecular weight compounds of taurine, ornithine and carnosine from new born calf liver. Zhongguo ying yong sheng li xue za zhi = Zhongguo yingyong shenglixue zazhi = Chin. J. Appl. Physiol..

[17] Serafeim A., Grafton G., Chamba A., Gregory C.D., Blakely R.D., Bowery N.G., Barnes N.M., Gordon J. (2002). 5-Hydroxytryptamine drives apoptosis in biopsylike Burkitt lymphoma cells: reversal by selective serotonin reuptake inhibitors. Blood.

[18] Koe B.K., Weissman A., p-Chlorophenylalanine (1966). A specific depletor of brain serotonin. J. Pharmacol. Exp. Therapeut..

[19] Jindo T., Wine R.N., Li L.H., Chapin R.E. (2001). Protein kinase activity is central to rat germ cell apoptosis induced by methoxyacetic acid. Toxicol. Pathol..

[20] Quash G., Roch A.M., Chantepie J., Michal Y., Fournet G., Dumontet C. (1995). Methional derived from 4-methylthio-2-oxobutanoate is a cellular mediator of apoptosis in BAF3 lymphoid cells. Biochem. J..

[21] Pekcetin C., Kiray M., Ergur B.U., Tugyan K., Bagriyanik H.A., Erbil G., Baykara B., Camsari U.M. (2009). Carnosine attenuates oxidative stress and apoptosis in transient cerebral ischemia in rats. Acta Biol. Hung..

[22] Georgakopoulou E.A., Achtari M.D., Achtaris M., Foukas P.G., Kotsinas A. (2012). Oral lichen planus as a preneoplastic inflammatory model. J. Biomed. Biotechnol..

[23] Zhang Y., Lin M., Zhang S., Wang Z., Jiang L., Shen J., Bai J., Gao F., Zhou M., Chen Q. (2008). NF-kappaB-dependent cytokines in saliva and serum from patients with oral lichen planus: a study in an ethnic Chinese population. Cytokine.

[24] Tsai S.J., Kuo W.W., Liu W.H., Yin M.C. (2010). Antioxidative and anti-inflammatory protection from carnosine in the striatum of MPTP-treated mice. J. Agric. Food Chem..

[25] Hope W.C., Patel B.J., Fiedler-Nagy C., Wittreich B.H. (1990). Retinoids inhibit phospholipase A2 in human synovial fluid and arachidonic acid release from rat peritoneal macrophages. Inflammation.

[26] Maling H.M., Webster M.E., Williams M.A., Saul W., Anderson W. (1974). Inflammation induced by histamine, serotonin, bradykinin and compound 48-80 in the rat: antagonists and mechanisms of action. J. Pharmacol. Exp. Therapeut..

[27] Ergun S., Trosala S.C., Warnakulasuriya S., Ozel S., Onal A.E., Ofluoglu D., Guven Y., Tanyeri H. (2011). Evaluation of oxidative stress and antioxidant profile in patients with oral lichen planus. J. Oral Pathol. Med. : Off. Publ. Int. Assoc. Oral Pathol. Am. Acad. Oral Pathol..

[28] Ali S.N., Ansari F.A., Mahmood R. (2016). Ameliorative effect of N-acetyl cysteine and taurine against sodium chlorate-induced oxidative stress in human erythrocytes. Free Radic. Biol. Med..

[29] Dutra F., Bechara E.J. (2004). Aminoacetone induces iron-mediated oxidative damage to isolated rat liver mitochondria. Arch. Biochem. Biophys..

[30] Francisco-Marquez M., Aguilar-Fernández M., Galano A. (2016). Anthranilic acid as a secondary antioxidant: implications to the inhibition of OH production and the associated oxidative stress. Comput. Theor. Chem..

[31] Koriem K.M., Fathi G.E., Salem H.A., Akram N.H., Gamil S.A. (2013). Protective role of pectin against cadmium-induced testicular toxicity and oxidative stress in rats. Toxicol. Mech. Methods.

[32] Lugo-Huitron R., Blanco-Ayala T., Ugalde-Muniz P., Carrillo-Mora P., Pedraza-Chaverri J., Silva-Adaya D., Maldonado P.D., Torres I., Pinzon E., Ortiz-Islas E., Lopez T., Garcia E., Pineda B., Torres-Ramos M., Santamaria A., La Cruz V.P. (2011). On the antioxidant properties of kynurenic acid: free radical scavenging activity and inhibition of oxidative stress. Neurotoxicol. Teratol..

[33] Pena-Silva R.A., Miller J.D., Chu Y., Heistad D.D. (2009). Serotonin produces monoamine oxidase-dependent oxidative stress in human heart valves. Am. J. Physiol. Heart Circ. Physiol..

[34] Holmstrup P., Dabelsteen E. (1979). Changes in carbohydrate expression of lichen planus affected oral epithelial cell membranes. J. Invest. Dermatol..

[35] Lowe N.J., Cudworth A.G., Clough S.A., Bullen M.F. (1976). Carbohydrate metabolism in lichen planus. Br. J. Dermatol..

[36] Dequattro V., Wybenga D., Von S., Brunjes S. (1964). Determination of urinary 3,4-dihydroxymandelic acid. J. Lab. Clin. Med..

[37] Kandagal S., Shenai P., Chatra L., Ronad Y., Kumar M. (2011). Effect of stress on oral mucosa. Brunnenbau,au Wasserw. Rohrleitungsbau.

[38] Burkhart N.W., Burker E.J., Burkes E.J., Wolfe L. (1996). Assessing the characteristics of patients with oral lichen planus. JADA (J. Am. Dent. Assoc.) (1939).

[39] Porte D. (1967). A receptor mechanism for the inhibition of insulin release by epinephrine in man. J. Clin. Invest..

[40] Porte D., Williams R.H. (1966). Inhibition of insulin release by norepinephrine in man. Science (New York, N.Y.).

[41] Dover E., Huang M., Douillet C., Stýblo M. (2016). Arsenite and methylarsonite impair glucose-stimulated insulin secretion through inhibition of mitochondrial respiration and calcium influx. Toxicol. Lett..

[42] Gelato M.C. (2003). Insulin and carbohydrate dysregulation. Clin. Infect. Dis.: Off. Publ. Infect. Dis. Soc. Am..

[43] Kalkur C., Sattur A.P., Guttal K.S. (2015). Role of depression, anxiety and stress in patients with oral lichen planus: a pilot study. Indian J. Dermatol..

[44] Vallejo M.J., Huerta G., Cerero R., Seoane J.M. (2001). Anxiety and depression as risk factors for oral lichen planus. Dermatology (Basel, Switzerland).

[45] Song C., Lin A., Bonaccorso S., Heide C., Verkerk R., Kenis G., Bosmans E., Scharpe S., Whelan A., Cosyns P., de Jongh R., Maes M. (1998). The inflammatory response system and the availability of plasma tryptophan in patients with primary sleep disorders and major depression. J. Affect. Disord..

[46] Smriga M., Kameishi M., Uneyama H., Torii K. (2002). Dietary L-lysine deficiency increases stress-induced anxiety and fecal excretion in rats. J. Nutr..

[47] Adamo D., Ruoppo E., Leuci S., Aria M., Amato M., Mignogna M.D. (2015). Sleep disturbances, anxiety and depression in patients with oral lichen planus: a case-control study. J. Eur. Acad. Dermatol. Venereol. : JEADV.

[48] Schwarz E., Whitfield P., Nahnsen S., Wang L., Major H., Leweke F.M., Koethe D., Lio P., Bahn S. (2011). Alterations of primary fatty acid amides in serum of patients with severe mental illness. Front. Biosci. (Elite edition).

[49] Akram M. (2014). Citric acid cycle and role of its intermediates in metabolism. Cell Biochem. Biophys..

